# Phase unwrapping with a rapid opensource minimum spanning tree algorithm (ROMEO)

**DOI:** 10.1002/mrm.28563

**Published:** 2020-10-26

**Authors:** Barbara Dymerska, Korbinian Eckstein, Beata Bachrata, Bernard Siow, Siegfried Trattnig, Karin Shmueli, Simon Daniel Robinson

**Affiliations:** ^1^ Department of Medical Physics and Biomedical Engineering University College London London United Kingdom; ^2^ High Field MR Center, Department of Biomedical Imaging and Image‐Guided Therapy Medical University of Vienna Vienna Austria; ^3^ Christian Doppler Laboratory for Clinical Molecular MR Imaging Medical University of Vienna Vienna Austria; ^4^ Magnetic Resonance Imaging The Francis Crick Institute London United Kingdom; ^5^ Centre for Advanced Imaging University of Queensland Australia; ^6^ Department of Neurology Medical University of Graz Graz Austria

**Keywords:** distortion correction, fMRI, MRI phase, multi‐echo, phase unwrapping, QSM

## Abstract

**Purpose:**

To develop a rapid and accurate MRI phase‐unwrapping technique for challenging phase topographies encountered at high magnetic fields, around metal implants, or postoperative cavities, which is sufficiently fast to be applied to large‐group studies including Quantitative Susceptibility Mapping and functional MRI (with phase‐based distortion correction).

**Methods:**

The proposed path‐following phase‐unwrapping algorithm, ROMEO, estimates the coherence of the signal both in space—using MRI magnitude and phase information—and over time, assuming approximately linear temporal phase evolution. This information is combined to form a quality map that guides the unwrapping along a 3D path through the object using a computationally efficient minimum spanning tree algorithm. ROMEO was tested against the two most commonly used exact phase‐unwrapping methods, PRELUDE and BEST PATH, in simulated topographies and at several field strengths: in 3T and 7T in vivo human head images and 9.4T ex vivo rat head images.

**Results:**

ROMEO was more reliable than PRELUDE and BEST PATH, yielding unwrapping results with excellent temporal stability for multi‐echo or multi‐time‐point data. It does not require image masking and delivers results within seconds, even in large, highly wrapped multi‐echo data sets (eg, 9 seconds for a 7T head data set with 31 echoes and a 208 × 208 × 96 matrix size).

**Conclusion:**

Overall, ROMEO was both faster and more accurate than PRELUDE and BEST PATH, delivering exact results within seconds, which is well below typical image acquisition times, enabling potential on‐console application.

## 1 INTRODUCTION

The complex signal in MRI can be divided into two constituents: magnitude (*M*) and phase (*θ*). The MRI phase is proportional to local deviations in the static magnetic field, ΔB_0_ (Hz), through the relation $\theta ∼2\pi TE\;\cdot \;\Delta B_{0}$. Knowledge of ΔB_0_ can be used to correct image distortions,^1^, ^2^ visualize veins and microbleeds using Susceptibility Weighted Imaging (SWI),^3^ assess iron‐rich tissues or calcifications through Quantitative Susceptibility Mapping (QSM),^4^ and to estimate blood flow^5^ or temperature changes in tissue.^6^ The measured phase, φ, is a projection of the true phase *θ* into the 2π range. This gives rise to abrupt changes (ie, wraps), which do not represent the spatial and temporal continuity of *θ* within the object and require unwrapping.

A questionnaire completed by 46 participants at the Fifth International Workshop on MRI Phase Contrast and Quantitative Susceptibility Mapping in South Korea (September 2019) indicated that 84.8% of participants use Laplacian unwrapping^7^ in their work, 32.6% use PRELUDE,^8^ 30.4% use BEST PATH,^9^ and 2.2% use Graph‐Cut^10^ (unpublished results reported by Prof. Peter C.M. van Zijl of Johns Hopkins University). Laplacian unwrapping is the most robust method currently available, providing globally smooth phase results (ie, no abrupt jumps) within seconds, even for large data sets with low SNR, explaining its popularity in QSM. However, it does not yield exact results for *θ*,^11^ which makes it unsuitable for applications such as distortion correction, flow, or temperature measurements (see Supporting Information Figure S1). Moreover, Laplacian unwrapping introduces large phase variations around regions with sharp phase changes, such as veins, which corrupt QSM results around these structures.^12^, ^13^, ^14^, ^15^, ^16^ PRELUDE and BEST PATH are the methods of choice when exact phase results are desired. They assume that phase changes between voxels that exceed π are indicative of wraps. PRELUDE belongs to the class of region‐growing spatial unwrapping approaches which divide the volume into wrapless regions (ie, groups of contiguous voxels containing ranges of values that are less than π) and assess phase changes at the borders between them. The PRELUDE algorithm is relatively robust and considered to be the gold standard,^11^ but it can take several hours or even days to unwrap large data sets with challenging phase topographies. A substantial reduction in computation time has been achieved using a recently developed method based on PRELUDE, called SEGUE,^17^ by simultaneously unwrapping and merging multiple regions. However, SEGUE can still take more than 10 minutes to unwrap more challenging data sets (eg, 17:35 minutes ± 9:26 minutes using a 3.5‐GHz processor for images acquired at 3T with matrix size = 220 × 220 × 240 and TE = 18.9 ms), making potential on‐console implementation impractical. Path‐following approaches, such as BEST PATH,^9^ usually provide solutions within seconds, even for highly wrapped images with large matrix sizes.^12^ This can be particularly useful in large studies, including functional MRI, in which hundreds of 3D image volumes are often acquired per subject. Path‐following algorithms compare the phase in adjacent voxels, beginning at one location and proceeding to neighboring voxels in an order dictated by the reliability of the information in the voxels and how well they are connected (ie, a quality map). BEST PATH is rapid but more prone to errors than PRELUDE, especially in regions where a corresponding magnitude image has low SNR. For a comprehensive comparison of phase‐unwrapping algorithms, we refer the reader to Robinson et al^12^ and Ghiglia and Pritt.^18^

To overcome the shortcomings of the exact phase‐unwrapping algorithms currently available, we propose a new path‐following algorithm called ROMEO: Rapid Opensource Minimum spanning treE algOrithm. This algorithm (1) uses up to three measures of the quality of connections between voxels, or weights, calculated from phase and magnitude information to provide improved unwrapping paths compared with BEST PATH, (2) provides computationally efficient bookkeeping of quality values and respective voxel edges, and (3) offers single‐step unwrapping of a fourth dimension (echo or time). We tested ROMEO’s performance against PRELUDE and BEST PATH in simulated topographies, challenging human head images acquired at 3T and 7T, and rat head images at 9.4T. Source code in the Julia^19^ programming language, compiled versions for Linux and Windows (easily executable using command line or *MATLAB* [MathWorks, Natick, MA]) and the data sets used in this study are publicly available (see Data Availability Statement).

## 2 METHODS

### 2.1 The ROMEO algorithm

It is important for the accuracy of a path‐based phase‐unwrapping method that the unwrapping proceeds along a path connecting reliable (albeit wrapped) voxels, as an error is likely to be introduced when an unreliable voxel (eg, noise value) is encountered. In common with many phase‐unwrapping methods, we draw on graph theory concepts to determine the optimum path. The edges connecting voxels are assigned weights, which indicate the reliability of the connection between them. Each of the many possible networks connecting all of the voxels in the image constitutes a spanning tree, and each spanning tree is associated with a weight that is the sum of all the weights of the edges in the tree. The minimum spanning tree is the spanning tree with the smallest weight: essentially the path connecting all voxels which includes the least unreliable connections between them.

The weights assigned to edges may consist of multiple contributions. The ROMEO algorithm uses up to three weights, which are multiplied together to yield a map of the “quality” of connection between neighboring voxels for each of the three principal directions (x, y, and z), here called a quality map. The unwrapping process is guided through the 3D phase data by this quality map, starting at the seed voxel; that with an edge with the highest quality value. For computational efficiency, the real‐valued quality values are converted into integer cost values that are sorted into a bucket priority queue^20^: a sequence of non‐negative integers, each of which has a “priority” associated with it: the cost value ranking. This effectively creates a minimum spanning tree of the cost values of the edges during the unwrapping process according to the Prim‐Jarník algorithm.^21^ The ROMEO algorithm was written in the open‐source programming language Julia,^19^ which has a syntax of similar simplicity to that of Python and a speed similar to the C‐based languages.

The ROMEO weights are defined in the range [0; 1] with “good” weights (those indicating well‐connected voxels) being close to 1, which allows easy combination of all or only some of the weights through multiplication. The three weights, calculated in each direction (x, y, and z), are defined as follows:

1. Spatial Phase Coherence weight:

(1) $$W_{\left(i,j,t\right)}^{\varphi ,Spat}=1‐\left|\frac{\Omega \left(\varphi_{i,t}‐\varphi_{j,t}\right)}{\pi }\right|,$$

where Ω is a wrapping operator; *φ_i,t_* and *φ_j,t_* are measured phases at two adjacent spatial locations, *i* and *j*; and $W_{\left(i,j,t\right)}^{\varphi ,Spat}$ is the spatial weight of edge (*i, j*), all at the same time point *t*.

- Temporal Phase Coherence weight:

(2) $$W_{\left(i,j,t\right)}^{\varphi ,Temp}=\text{max}\left(0,1‐\left|\Omega \left(\varphi_{i,t‐1}‐\varphi_{j,t‐1}\right)‐\Omega \left(\varphi_{i,t}‐\varphi_{j,t}\right)\;\cdot \;TE_{t‐1}/TE_{t}\right|\right),$$

where *t =* 2 and phase values for the first and second echo (TE_1_, TE_2_) are chosen as the default for the calculation of the temporal coherence weight, $W_{\left(i,j,t\right)}^{\varphi ,Temp}$. It is possible to change *t* if desired.

- Magnitude Coherence weight:

(3) $$W_{\left(i,j,t\right)}^{M}={\left(\text{min}\left(M_{i,t},M_{j,t}\right)/\text{max}\left(M_{i,t},M_{j,t}\right)\right)}^{2},$$

where *M_i,t_* and *M_j,t_* are the magnitudes at two adjacent spatial locations *i* and *j* at time *t*.

**FIGURE 1:**
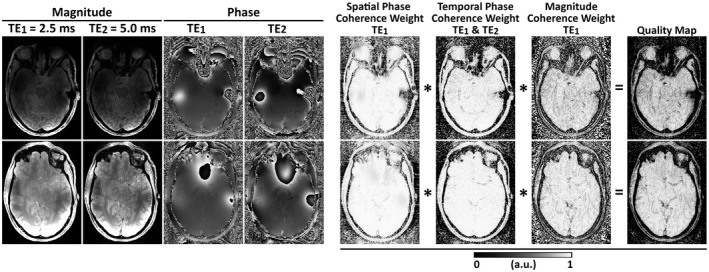
Example of maps of the three ROMEO weights for the x‐direction (left–right): Magnitude and phase images at the first two TEs, Spatial Phase Coherence, Temporal Phase Coherence, and Magnitude Coherence weights. Multiplication of these weights defines the final quality map for the x‐direction. Two axial slices are shown from data acquired at 7T with the 3D gradient‐echo (GRE) sequence parameters found in Table 1. These maps were calculated for the first echo using the magnitude image acquired at TE_1_ and phase images acquired at TE_1_ and TE_2_. The weights and quality values close to 1 correspond to good voxel connections; values close to 0 are weakly connected and are unwrapped last. Analogous weights are calculated for the y and z directions

Weight 2 is only used for multi‐echo or multi‐time‐point data and can be omitted. Weight 3 is used if magnitude data are available. An example of the three weights for the x‐direction (left–right) for a 7T 3D gradient‐echo (GRE) data set is shown in Figure 1.

The product of the weights for each direction yields a quality map for each direction. For computational efficiency and reduced memory usage, the real valued quality map between 0 and 1 is transformed into integer cost values between 255 and 1 (ie, $cost=\text{max}\left(round\left(255\;\cdot \;\left(1‐quality\right)\right),1\right)$. An integer cost value of 1 corresponds to the best connection and a cost value of 255 to the worst. The special case of a cost value of 0 denotes no connection between voxels (eg, the border of a mask). In this case, the corresponding phase values are not included in the priority queue, which effectively stops the unwrapping process in that direction. The range of values from 0 to 255 was chosen, as it can be stored efficiently as an 8‐bit unsigned integer (2^8^ = 256) and represents the range of original real‐valued quality values with sufficient accuracy to avoid changing the unwrapped result. Cost values derived from the quality map and corresponding voxel edge locations along the three different axes are passed into the bucket priority queue. The priority queue initially contains six cost values surrounding the seed voxel (in directions ‐x, x, ‐y, y, ‐z, z). The smallest value in the queue is identified together with the corresponding edge connecting the seed voxel (voxel 1) with its neighbor (voxel 2). If there is a phase jump > π between these voxels, 2πn is subtracted from the phase of voxel 2 according to $\theta_{2,t}=\varphi_{2,t}‐2\pi n=\varphi_{2,t}‐2\pi *round\left(\frac{\varphi_{2,t}‐\theta_{1,t}}{2\pi }\right)$, where *φ*_2,_ *_t_* is the wrapped phase measured in voxel 2, *θ*_1,_ *_t_* is the phase in voxel 1, and *θ*_2,_ *_t_* is the unwrapped phase in voxel 2, all at a given time point *t*. Voxel 2 is subsequently marked as having been visited. New values are added to the queue, including the connections between voxel 2 and all of its neighbors not yet visited by the algorithm. When a new edge is drawn from the queue, a check is performed to see whether the voxels connected by the edge have both been visited: If they have, this edge is removed from the queue. The search for the minimal cost value and the unwrapping process are repeated iteratively until all voxels have been visited (Supporting Information Figure S2).

By default, ROMEO calculates weights only for a single 3D volume in multi‐echo or multi‐time‐point data—a template phase volume—and uses the unwrapped result from this template, *θ_i,t_*, to unwrap the neighboring volumes, *φ_i,t_*_±1_, assuming an approximately linear phase evolution in time:

(4) $$\theta_{i,t\pm 1}=\varphi_{i,t\pm 1}‐2\pi *round\left(\frac{\varphi_{i,t\pm 1}‐\theta_{i,t}*\frac{TE_{i,t\pm 1}}{TE_{i,t}}}{2\pi }\right).$$

This accelerates the unwrapping process substantially by avoiding the recalculation of the weights for each single volume and improves the stability of the unwrapping results over the echoes or time points. By default, *t* = 2 is specified as the template phase volume, because *t* = 1 tends to be more affected by flow effects (in multi‐echo GRE) or is acquired before the longitudinal magnetization reaching a steady state (in EPI time series). The template phase volume can be changed if necessary. In specific cases, when large motion occurs between the time points or the assumption of linear phase evolution is not fulfilled, individual phase unwrapping can be applied with the calculation of weights and spatial unwrapping for each volume.

### 2.2 Data sets

**FIGURE 2:**
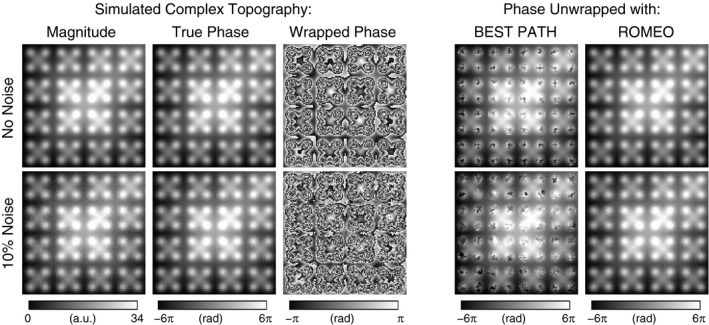
BEST PATH and ROMEO phase‐unwrapping results for the simulated complex topography at TE = 10 ms. PRELUDE failed to deliver results within 38 days. BEST PATH generated a large number of errors for a topography with no noise (top row)—all voxels of which were correctly unwrapped with ROMEO. The addition of 10% noise (bottom row) saw a substantial increase in the number of errors with BEST PATH and the emergence of the first few incorrectly unwrapped voxels in ROMEO (bottom row), showing ROMEO to define more effective, noise‐robust paths through the image. Results are evaluated over the whole image volumes and all echoes in Supporting Information Table S1

To provide a ground‐truth phase, *θ*, for a complicated pattern of wraps in φ, a complex topography was simulated as in Robinson et al^22^ and Karsa and Shmueli^17^ (difficulty 4), with matrix size 256 × 256 × 256 and TEs = [4, 8, 10] ms, with the addition of no noise and 10% noise, expressed as a percentage of the phase at each TE. Example magnitude and phase images of this topography at TE = 10 ms are shown in Figure 2.

Measured phase maps (with no ground‐truth *θ*) were also examined: in vivo human head MRI acquisitions at 3T and 7T (Siemens MAGNETOM; Siemens Healthineers, Erlangen, Germany) and ex vivo rat head images acquired at 9.4T (Bruker BioSpec 94/20 USR; Bruker, Ettlingen, Germany) with sequence parameters listed in Table 1.

**TABLE 1:** Data sets used for the assessment of ROMEO unwrapping accuracy and computational speed

Figure No.	B_0_	Head RF coil, No. of channels	Sequence	No. of scans	TE (ms)	TR (ms)	Matrix size	Resolution (mm)	Receiver bandwidth (Hz/pixel)	Flip angle (°)
2	Simulation, N/A				4, 8, 10	N/A	256 × 256 × 256	Simulation, N/A		
3, 7	3T	64	2D GRE	1	5, 10, 16, 21, 26, 31	4370	144 × 144 × 84	1.5 × 1.5 × 1.5	270	90
3	3T	64	2D EPI	3	31, 35, 40 (three scans)	8680	144 × 144 × 84	1.5 × 1.5 × 1.5	1655	90
1, 4, 7	7T	32	3D GRE	1	TE_1_/dTE/TE_31_ = 2.5/2.5/77.50	80	208 × 208 × 96	1.0 × 1.0 × 1.0	859	15
5, S3	7T	32	2D EPI	5 patients (57 volumes each)	22	2500	128 × 128 × 40	1.7 × 1.7 × 3.0	1447	75
6, 7	9.4T	4	3D GRE	1	TE_1_/dTE/TE_12_ = 1.4/1.4/16.8	22	150 × 125 × 175	0.2 × 0.2 × 0.2	1333	8.7

Human measurements were approved by the Ethics Committee of the Medical University of Vienna, and all participants provided written, informed consent. All human data sets were acquired from healthy volunteers except for five 7T EPI time series (57 volumes) from a previous study,^23^ which were collected from 4 patients with brain tumors and 1 patient with a developmental venous anomaly.

### 2.3 Data analysis

All of the data were acquired with multichannel coil arrays. Separate channels were combined using ASPIRE^24^ for multi‐echo GRE data and using the coil combination described in Dymerska et al^25^ for the single‐echo EPI data. Combined phase images were unwrapped using compiled PRELUDE from the FSL toolbox (version 5.0.11; https://fsl.fmrib.ox.ac.uk/fsl/fslwiki/FSL) written in C++,^8^ BEST PATH^9^ programmed in C, and ROMEO written in the Julia programming language. Their performance was compared with respect to unwrapping accuracy and computational speed. All of the calculations were performed on a PC with an Intel Xeon W‐2125 4.0‐GHz processor, 64 GB RAM, and an Ubuntu Linux 16.04 operating system.

All ROMEO results were obtained using magnitude and phase images as well as template unwrapping as described in section 2.1. Images were additionally masked to obtain PRELUDE results in a feasible time. Unmasked and masked measured data were analyzed for BEST PATH and ROMEO. Masking was performed with the FSL Brain Extraction Tool^26^ for in vivo data other than the 3T EPI, in which the SPM Segment Toolbox (SPM12; https://www.fil.ion.ucl.ac.uk/spm/) was used, as the Brain Extraction Tool produced masks that did not match the image well. Ex vivo rat head images were masked using magnitude image thresholding.

A qualitative comparison of the results was performed using MRIcro (https://www.mccauslandcenter.sc.edu/crnl/mricro) and FSLeyes (https://fsl.fmrib.ox.ac.uk/fsl/fslwiki/FSLeyes), and selected slices with substantial differences between the three phase unwrapping methods are presented in section 3.1.

For the simulated data, quantitative comparison was performed by calculating the percentage of unwrapped values that were different from the ground truth. Although for in vivo measurement there is inherently no ground truth available, a reliable estimate of the true phase, or temporal reference image, can be obtained for multi‐echo data if the first TE is short and the TE difference between consecutive echoes is small, ensuring small and approximately linear phase evolution between the echoes. This was found to be the case for the multi‐echo GRE data at 3T, 7T, and 9.4T. For the first TE, the temporal reference image was merged from the results of the three methods analyzed here: Voxels were only included if their unwrapped phase values were the same for all three unwrapping methods. This meant that for the 3T , 7T, and 9.4T GRE data, respectively, 99%, 98%, and 89% of the voxels within the brain mask were included in the temporal reference image. The temporal reference for subsequent echoes was calculated by assuming approximately linear phase evolution over time using Equation 4. Phase‐unwrapping errors were calculated as the difference between the unwrapped phase obtained using a given method at a given TE and the temporal reference at the same TE. Histograms showing the number of voxels with 2πn errors for all three methods analyzed were plotted (see Figure 7). The percentage of voxels with unwrapped phase values different than the temporal reference within the mask is listed in Table.

For EPI at 3T and 7T, calculation of a temporal reference as described previously was not possible due to the inherently long minimum TEs of the EPI acquisitions. These images were assessed visually, using MRIcro and FSLeyes. For the single‐echo 7T EPI time‐series data, temporal mean SD images were calculated throughout the brain mask to investigate regions where unwrapping errors were different at various time points (Figure 5). The SD of the estimated field map ($\Delta B_{0}\left(\mathrm{TE}\right)=\frac{\theta \left(\mathrm{TE}\right)}{\mathrm{TE}}$) was calculated for the 9.4T GRE data set with 12 echoes (Figure 6).

## 3 RESULTS

### 3.1 Comparison of unwrapping accuracy among PRELUDE, BEST PATH, and ROMEO

Figure 2 shows the BEST PATH and ROMEO unwrapping results for the simulated topography data at TE = 10 ms. Results over the whole image volumes and for all TEs are listed in Supporting Information Table S1. The PRELUDE algorithm failed to complete unwrapping within 38 days (912 hours). The BEST PATH method delivered accurate results only for TE = 4 ms, with errors between 2% and 3% (depending on noise) for the more complex topography at TE = 8 ms and between 7.5% and 9% for the highly wrapped image corresponding to TE = 10 ms. The ROMEO algorithm provided more accurate results, with a small number of errors (0.1%) only for the longest TE data with noise added.

**FIGURE 3:**
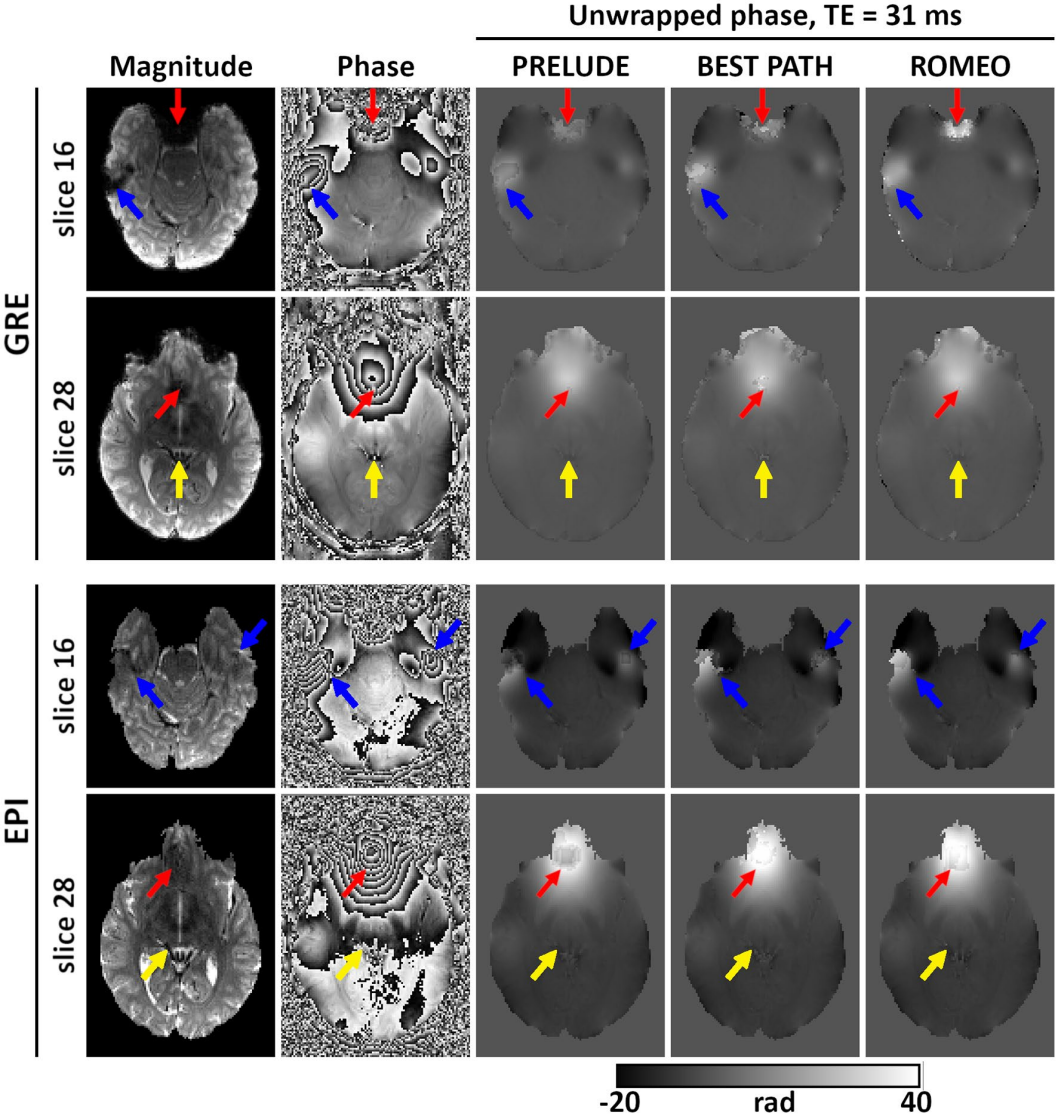
Unwrapping results for the 3 T GRE and EPI scans at TE = 31 ms. Magnitude and phase images (wrapped and unwrapped) are shown for two slices. Red arrows point to the regions close to the sinuses; blue arrows point to open‐ended fringe lines close to the ear canals; and yellow arrows point to vessels affected by low signal and unwrapping errors. Overall, ROMEO results had the smallest number of voxels with residual phase wraps

Unwrapping results for the 3T GRE and EPI data set with TE = 31 ms are presented in Figure 3. Two slices with visible differences among PRELUDE, BEST PATH, and ROMEO are shown. Unwrapping errors occurred in all methods close to the sinuses (red arrows), where BEST PATH shows the largest errors. In both the GRE and EPI phase, an open‐ended fringe line is clearly visible in the wrapped phase close to the left ear canal, where the magnitude signal approaches the noise level (blue arrows). In ROMEO, the extent of the unwrapping error in this region was limited to a few voxels. In PRELUDE and BEST PATH, the size of the region affected increased with TE (not shown). A residual wrap also occurred in the vein of Galen in the BEST PATH result (see Figure 3 GRE, yellow arrows). Unwrapping differences among the three methods were also observed in a small number of voxels in other vessels (see Figure 3 EPI, yellow arrows). Regions affected by residual wraps were the smallest in ROMEO.

**FIGURE 4:**
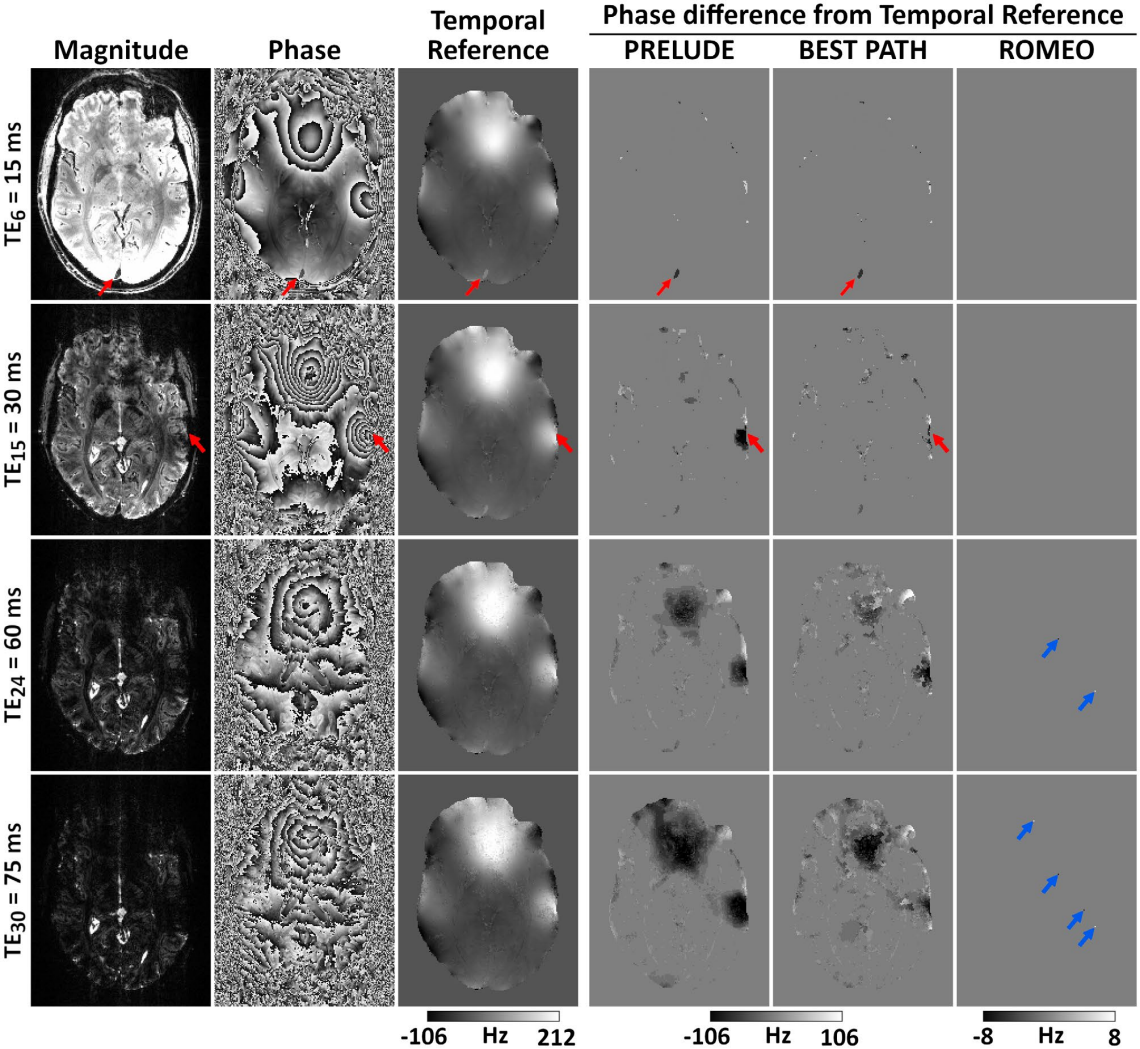
Unwrapping results for the 7T GRE data at four selected echoes (of 31). At shorter echoes (TE_6_ = 15 ms and TE_15_ = 30 ms), a few unwrapping differences between the PRELUDE or BEST PATH and the temporal reference phase occur at the brain edges, sagittal sinus, or ear canals (see red arrows). At longer echoes (TE_24_ = 60 ms and TE_30_ = 75 ms), large patches with unwrapping errors are visible in PRELUDE and BEST PATH results. There is no difference between the temporal reference phase and the ROMEO result at TE_6_ and TE_15_, and only a few voxels (marked by blue arrows) differ at longer echoes

**FIGURE 5:**
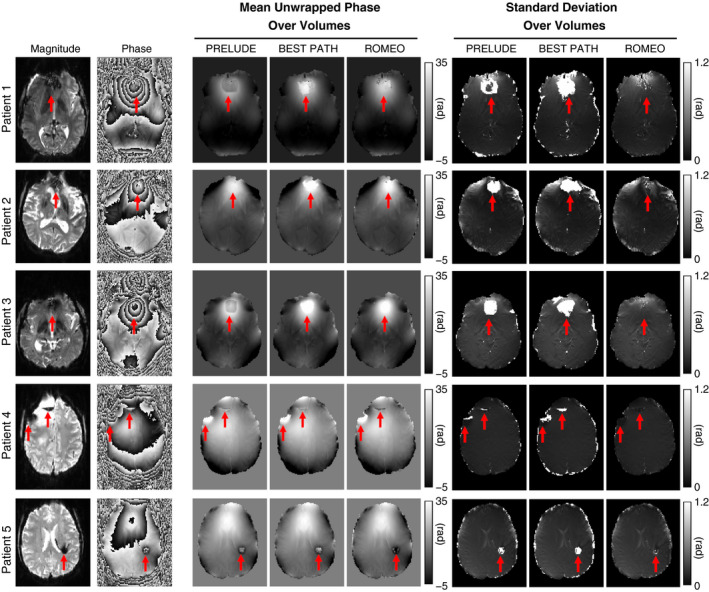
Unwrapping results for a 7T EPI time series with 57 volumes acquired in 4 patients with brain tumors and 1 patient (patient 5) with a developmental venous anomaly. The temporal mean and SD of the unwrapped phase are shown for all patients. The SD maps highlight residual phase wraps, which change for different time points. Red arrows highlight the largest errors. The ROMEO method outperformed PRELUDE and BEST PATH, yielding both fewer residually wrapped voxels and less temporal variation in the unwrapped phase over time points

Examples of phase‐unwrapping performance for PRELUDE, BEST PATH, and ROMEO at 7T are presented in Figure 4 for multi‐echo GRE and in Figure 5 for a single‐echo EPI time series. A central axial slice from the GRE data is shown at four selected TEs in Figure 4, starting with a relatively short TE (TE_6_ = 15 ms) and ending with a very long one (TE_30_ = 75 ms), where the signal in a large part of the image has decayed into noise. At TE_6_ = 15 ms, small differences were observed at the brain boundaries and in the sagittal sinus (red arrows) between the temporal reference and the PRELUDE or BEST PATH results. At TE_15_ = 30 ms, slightly larger regions with unwrapping errors were observed in the PRELUDE and BEST PATH results, such as close to the auditory canals (red arrows). At later echoes, such as TE_24_ = 60 ms and TE_30_ = 75 ms, large patches of tissue were affected by phase‐unwrapping errors in both the PRELUDE and BEST PATH results, with larger regions of errors observed at longer TEs. No difference between the temporal reference and the ROMEO unwrapped phase is visible in this slice at TE_6_ and TE_15_, but differences in a few voxels are observed at TE_24_ and TE_30_ (blue arrows). Regions with a very low SNR, approaching the noise floor in the magnitude image, are noisy in both the temporal reference and the ROMEO unwrapped phase; otherwise, both show a coherent phase topography. Small differences between the two results are more apparent in the quantitative comparison of the methods in Figure 7 and Table 2.

**TABLE 2:** Percentage of erroneous voxels within the mask for all three phase‐unwrapping methods and second, middle, and last echo of the GRE acquisitions at 3T, 7T, and 9.4T

	TE (ms)	No. of erroneous voxels within the mask (%)		
		PRELUDE	BEST PATH	ROMEO
3T GRE	10.0	0.53	0.61	0.42
	21.0	1.74	1.92	0.42
	31.0	2.97	3.31	0.42
7T GRE	5.0	0.27	0.29	0.12
	40.0	5.70	5.40	0.12
	77.5	22.81	23.40	0.12
9.4T GRE	2.8	1.45	1.74	0.85
	9.8	14.50	14.00	0.86
	16.8	26.58	24.85	0.86

Note

Voxels with 2πn phase differences (where n is an integer) from the temporal reference were counted as erroneous (see section 2.3).

PRELUDE and BEST PATH results for the 7T EPI time series (57 volumes) were affected by global 2πn phase jumps between consecutive time points, numbering 0, 19, 0, 14, and 32 jumps, respectively, for patients 1 to 5 for PRELUDE and 17, 10, 18, 0, and 26 for BEST PATH. No phase jumps between time points were present in the ROMEO results. Therefore, global phase jumps in the PRELUDE and BEST PATH results were removed before the calculation of the temporal mean and SD of the unwrapped phase, which are shown in Figure 5. In patients 1, 2 and 3, extensive unwrapping errors occurred using PRELUDE and BEST PATH close to the sinuses, marked by the arrows. These errors changed in size at different time points, which contributed to the high values of the phase SD in these regions. The ROMEO unwrapping errors were substantially smaller and stable over time points, which is reflected in low SD values. In patients 4 and 5, the unwrapping errors occurred close to pathologies and were, again, less extensive for ROMEO than for PRELUDE or BEST PATH. The effect that these errors unwrapping EPI have on a dynamic distortion correction is illustrated in Supporting Information Figure S3.

**FIGURE 6:**
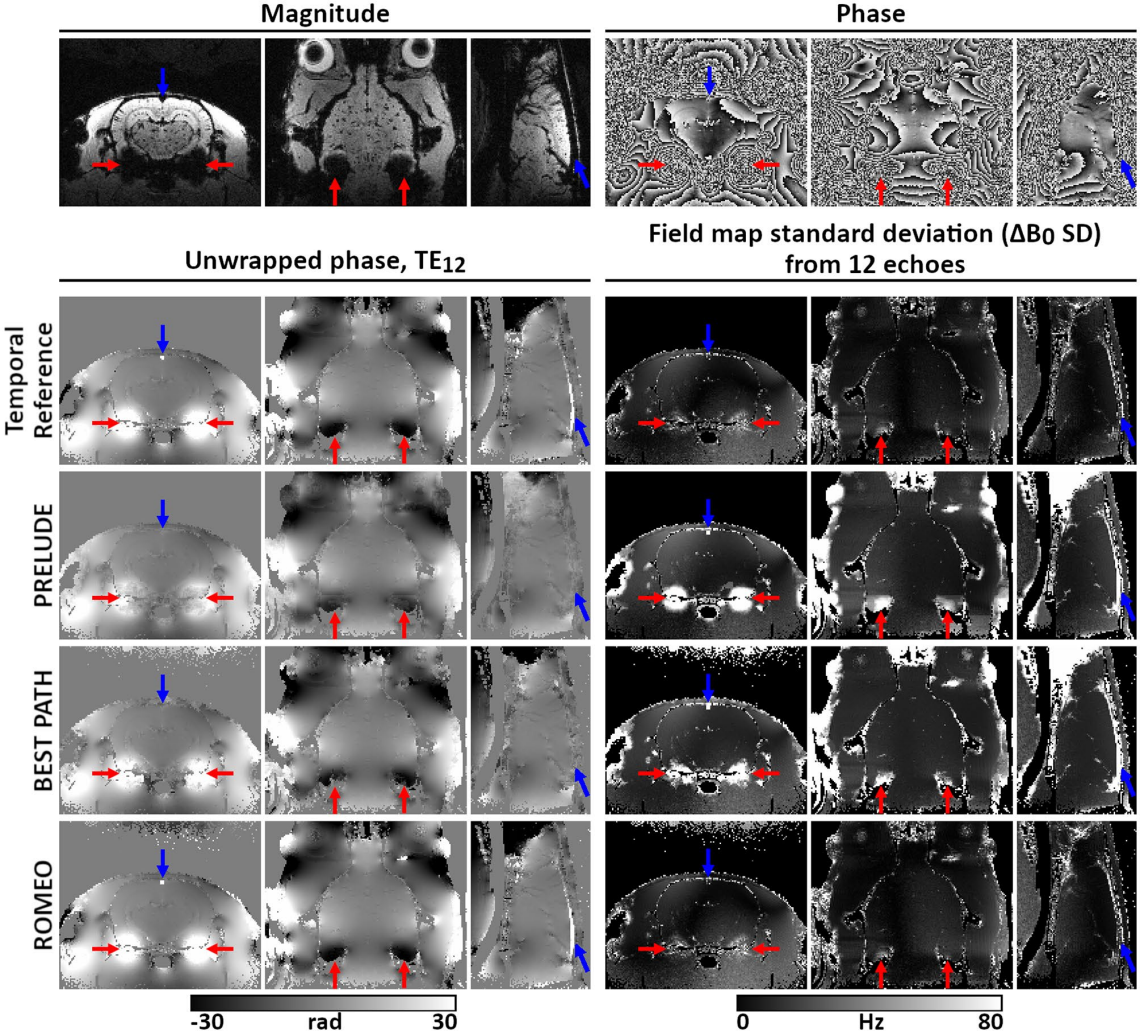
Unwrapping results for images of a rat brain acquired at 9.4 T using a multi‐echo GRE sequence (TE_1_/dTE/TE_12_ = 1.4/1.4/16.8 ms). The magnitude and unwrapped phase from the last echo (TE_12_) and the field‐map SD (ΔB_0_ SD) over all 12 echoes are shown in all three perpendicular planes. Differences between the methods are highlighted by red and blue arrows. The ROMEO unwrapping results are the most accurate, the most similar to the temporal reference, and the most stable over echoes

Figure 6 shows high‐resolution images of an ex vivo rat brain, acquired at 9.4 T using a multi‐echo GRE sequence. The ROMEO algorithm gave the most accurate phase‐unwrapping results, agreeing well with the temporal reference, and with very good stability over the echoes, as highlighted by the ΔB_0_ SD. Regions in PRELUDE and BEST PATH results marked by red arrows were affected by residual phase errors, which changed for different echoes (see corresponding ΔB_0_ SD maps). Blue arrows point to the superior sagittal sinus, which had a phase offset with respect to the surrounding tissue in both the temporal reference and ROMEO results, which was consistent for all echoes, as represented by the low ΔB_0_ SD values. The phase images unwrapped by PRELUDE and BEST PATH show similar offsets in the sagittal sinus, but only at some of the shorter TEs (five echoes in PRELUDE and seven in BEST PATH), which is reflected by high ΔB_0_ SD values in this large vein.

**FIGURE 7:**
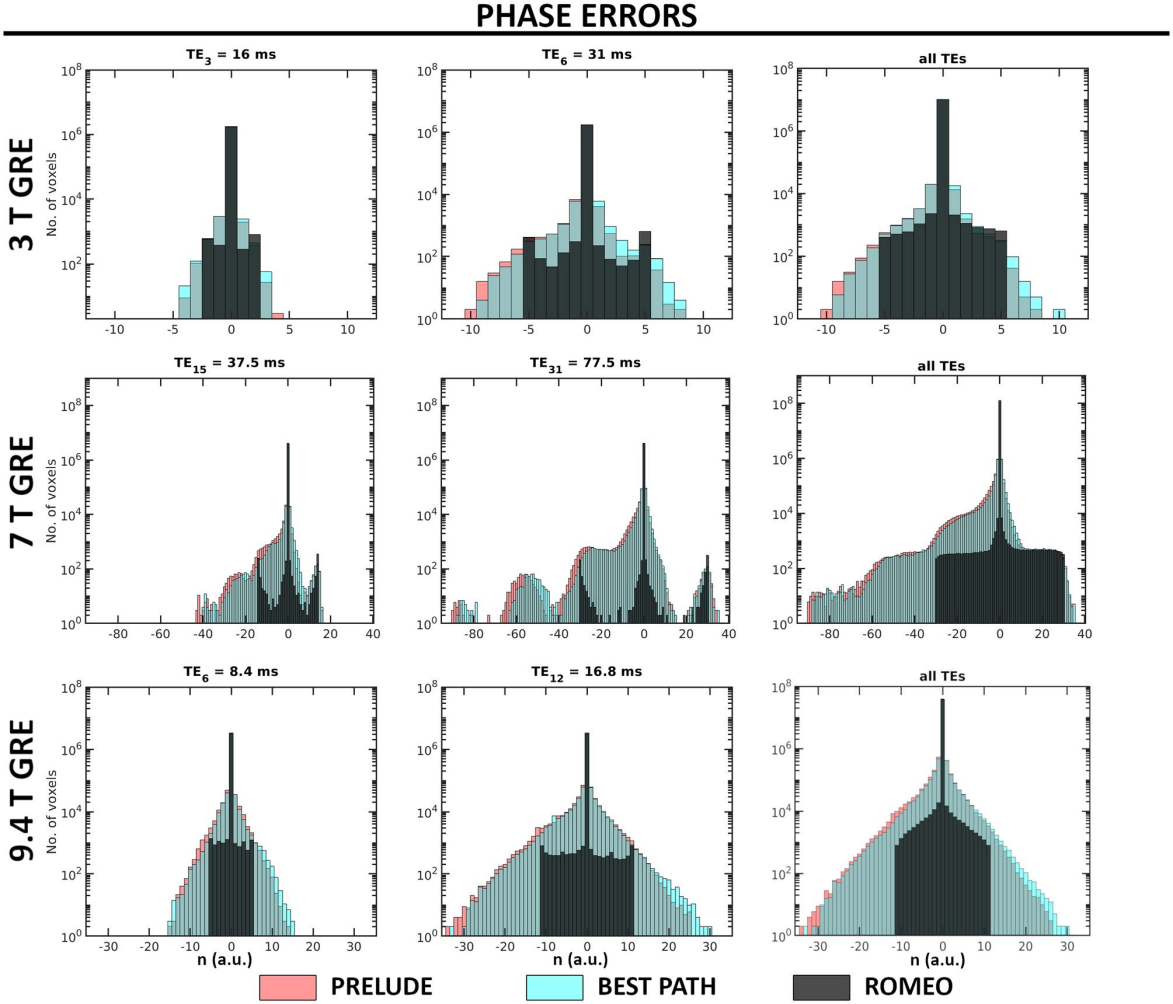
Histograms of phase errors in PRELUDE, BEST PATH, and ROMEO GRE results at all field strengths for the central and last TEs, and summed over all echoes (excluding TE_1_). Voxels with 2πn (where n is an integer) phase differences from the temporal reference phase were counted as erroneous (see section 2.3). The number of voxels is shown on a logarithmic scale

Histograms showing the number of voxels with 2πn phase errors (n is an integer) in the unwrapped GRE results at 3T, 7T and 9.4T, for the middle echo, the last echo, and over all echoes are presented in Figure 7. The PRELUDE and BEST PATH results show similar error spectra, which increase in amplitude and become broader at longer TEs. The number of erroneous voxels in ROMEO is substantially smaller than for the other unwrapping methods. This result is also highlighted in Table 2, where the percentage of erroneous voxels within the mask is listed for all methods and three selected echoes. For all methods, the number of erroneous voxels increased with the TE. For ROMEO results, this number was below 1% at all field strengths and all echoes. The PRELUDE and BEST PATH errors affected over 20% of the voxels at the longest echoes at 7 T and 9.4 T.

All of the described wrapped phase images were masked to obtain PRELUDE results in feasible times. For BEST PATH and ROMEO, no mask was required; therefore, unwrapping with no mask was also assessed. This yielded identical results, within masks, to this analysis. There was, however, a difference in computation time between executions with or without a mask, as described subsequently.

### 3.2 Comparison of computational speed among PRELUDE, BEST PATH, and ROMEO

The computation times of ROMEO in comparison to PRELUDE and BEST PATH are summarized in Table 3. PRELUDE unwrapping took from several minutes (14 minutes 36 seconds for 3T GRE) to several hours (128 hours 8 minutes 59 seconds for 7T GRE), and the unwrapping process failed to finish within 38 days for the simulated data set. BEST PATH took less than a minute for all masked data, and ROMEO took at most 20 seconds. ROMEO was generally faster than BEST PATH with the exception of 3T data sets, in which BEST PATH was faster by, at most, 4 seconds (see Table 3 for more details).

**TABLE 3:** Computation times for PRELUDE, BEST PATH, and ROMEO

Data set		Computation time (hh:mm:ss)		
		PRELUDE	BEST PATH	ROMEO
Simulation	Not masked	Not finished in 912 hours	00:00:38	**00:00:20**
3T GRE	Masked	00:14:36	**00:00:04**	00:00:06
	Not masked		00:00:07	00:00:07
3T EPI	Masked	00:32:25	**00:00:02**	00:00:06
	Not masked		00:00:04	00:00:07
7T GRE	Masked	128:08:59	00:00:48	**00:00:09**
	Not masked		00:01:31	00:00:11
7T EPI				
Patient 1	Masked	01:03:29	00:00:11	**00:00:06**
	Not masked		00:00:26	00:00:06
Patient 2	Masked	00:30:37	00:00:11	**00:00:06**
	Not masked		00:00:25	00:00:06
Patient 3	Masked	00:29:03	00:00:11	**00:00:06**
	Not masked		00:00:25	00:00:06
Patient 4	Masked	00:38:43	00:00:09	**00:00:06**
	Not masked		00:00:26	00:00:06
Patient 5	Masked	00:27:22	00:00:11	**00:00:06**
	Not masked		00:00:26	00:00:07
9.4T GRE	Masked	56:03:07	00:00:17	**00:00:08**
	Not masked		00:00:27	00:00:09

Note

The fastest times for each data set are in bold.

Using BEST PATH and ROMEO, data sets that were not masked took longer to unwrap than masked images. This difference was less prominent for ROMEO, with the same unwrapping times for masked and unmasked 7T EPI data sets (6 seconds). All three methods were memory‐efficient, with the maximum RAM use below 5 GB for data sets (magnitude and phase images) with size below 800 MB.

## 4 DISCUSSION

We have presented a new, rapid, and robust phase unwrapping technique—ROMEO—that is more reliable and faster than the two exact phase‐unwrapping algorithms most commonly used in MRI: PRELUDE and BEST PATH. Because the MRI signal is complex, magnitude information is available for every MRI scan, even if a study focuses exclusively on phase imaging. The ROMEO method includes information about the spatial coherence of the magnitude signal in the unwrapping operation. Combining this information with information on the phase’s spatial and temporal coherence creates a refined quality map that guides the unwrapping process through 3D data, starting with the most reliable voxels. This improves the unwrapping accuracy over BEST PATH, which uses a quality map based on only a single weight calculated from the second difference of the phase between the six nearest neighbors and the 20 diagonal neighbors of a given voxel. Moreover, ROMEO uses “template‐based” unwrapping with respect to a selected template volume when the data have a fourth dimension (eg, echoes or time points). This allows ROMEO to avoid introducing 2πn phase jumps between these echoes or time points and speeds up the unwrapping operation, as the weights and quality map are calculated only for one 3D template volume. Template unwrapping works accurately if the phase is proportional to TE (ie, linear for multi‐echo data, constant for single‐echo time‐series data) and no residual phase offsets are present (ie, *θ* ≈ 0 at TE = 0). We offer ROMEO version 3.1 (see Data Availability Statement) with the possibility to remove residual phase offsets using the MCPC‐3D‐S method.^24^ If the phase is nonlinear with time (eg, due to large motion), ROMEO offers the option of unwrapping each volume individually.

The phase‐unwrapping problem exists because it is not possible to measure the ground‐truth phase, which means that creating a reference image and performing a quantitative comparison of different phase‐unwrapping algorithms in vivo is challenging. The temporal reference was calculated with the assumption that the phase evolves approximately linearly over time, which is, for the purpose of assessing wraps, a reasonable approximation of the ground truth. It is only possible to calculate such a temporal reference from multi‐echo acquisitions with a short initial TE and small echo spacings. The first TE phase must be either free of wraps or unwrapped without errors by all of the methods under evaluation. In addition to a thorough qualitative comparison presented in Figures 2, 3, 4, 5, 6, we have also provided a quantitative analysis of unwrapping errors for all three phase‐unwrapping methods considered here (see Figure 7 and Table 2). We have calculated the percentage of voxels in each method with values different from the ground truth in simulation or from the temporal reference in measured data. The ROMEO method uses template unwrapping, a type of temporal unwrapping, which yielded results that agreed well with the temporal reference.

Path‐following methods, BEST PATH and ROMEO, were much faster than PRELUDE, a region‐growing method. The improved speed of ROMEO with respect to BEST PATH arises from template unwrapping as well as from efficient handling of values in the queue of voxels to be considered. The BEST PATH method uses the Kruskal algorithm to calculate the minimum spanning tree,^27^ using a heap as the priority queue, which has a runtime that depends on the number of voxel insertions into the queue, m, according to O(m log[m]). The ROMEO method uses the Prim‐Jarník algorithm^21^ with integer representation in a bucket priority queue, the runtime of which scales with O(m). Some of the speed differences may also come from the fact that the two methods were implemented in different programming languages (BEST PATH in C, ROMEO in Julia). The ROMEO method requires initialization of the Julia runtime before unwrapping, which contributes to the fact that ROMEO unwrapping was never faster than 6 seconds for the data presented here.

ROMEO took only a few seconds, even for very challenging examples such as 7T GRE images (9 seconds for masked images), whereas PRELUDE delivered results after about 128 hours and BEST PATH took 48 seconds. Although ROMEO was usually several seconds faster than BEST PATH (except in small 3T data sets), this is less relevant than the unwrapping accuracy. The ROMEO method showed fewer residual phase wraps than BEST PATH and PRELUDE in all of the analyzed cases. Additionally, ROMEO demonstrated superior phase‐unwrapping stability over time points or echoes, which was highlighted by phase or field‐map SD results. The PRELUDE and BEST PATH methods often showed a different distribution of residual wraps in problematic areas (eg, close to open‐ended fringe lines) at different time points, rendering large areas of the phase images unusable, and requiring post hoc global 2πn jump correction between adjacent volumes.

The unwrapping accuracy was independent of masking for BEST PATH and ROMEO, which highlights the redundancy of masking for these path‐following methods and allows time to be saved that is normally spent on the often‐fraught problem of mask generation. The PRELUDE algorithm only generated results when a mask was provided, but even then, calculation times were excessively long. As shown using simulated data, not masking PRELUDE inputs led to the algorithm failing to yield results even after many days. The quality maps calculated in ROMEO can be combined and thresholded to generate an object mask. This could be useful in applications requiring a mask such QSM, particularly for inhomogeneous images and non‐brain regions or phantoms, where commonly used methods such as the Brain Extraction Tool do not perform well. ROMEO is extremely flexible, as it has the option to output individual weights and the quality map (both combined over x, y, and z) as well as a mask.

There is substantial interest in using EPI sequences for phase imaging and QSM.^28^, ^29^, ^30^, ^31^, ^32^ Phase images from EPI acquisitions were generally more challenging to unwrap than those from GRE scans, because EPI has a lower SNR than GRE and is affected by other effects such as distortions in the phase‐encoding direction or stronger eddy currents. Of the three methods tested, ROMEO proved to be the most accurate and robust unwrapping algorithm for single‐echo EPI acquisitions.

Three weights were included in the ROMEO implementation discussed here. We offer the source code in the Julia programming language, which allows users to experiment with alternative weights for atypical MRI acquisitions or phase data sets acquired using other modalities such as optical or satellite radar interferometry.

We expect ROMEO to find applications in MRI phase imaging and QSM, especially in challenging cases such as at high fields, at long TEs, in highly accelerated data sets with low SNR, and close to air spaces or implants. The speed of ROMEO speed makes it feasible to use spatial phase unwrapping in real time on the MRI reconstruction computer, which could benefit large studies with hundreds or thousands of phase volumes, including functional MRI studies in which phase information can be used to correct distortions^25^, ^33^ and provide quantitative information about changes in blood susceptibility in functional QSM.^30^, ^31^, ^32^

## 5 CONCLUSIONS

We have developed a new path‐following phase‐unwrapping algorithm called ROMEO, which is more accurate and faster than PRELUDE and BEST PATH, yielding results within a few seconds even for highly wrapped data with large matrix sizes. The ROMEO algorithm does not require explicit masking and allows single‐step unwrapping of multi‐echo or multi‐time‐point data with excellent stability over volumes. Therefore, it is suitable for MRI studies in which phase unwrapping is challenging, such as at high fields, with implants or in large data sets including functional MRI studies.

## Supporting information


**FIGURE S1** A comparison of the path‐based method (ROMEO [rapid opensource minimum spanning tree algorithm]) and Laplacian unwrapping. The path‐based method restores the simulated ground‐truth phase from the wrapped phase, yielding exactly the true phase value in every voxel. The Laplacian method removes wraps but introduces phase offsets and background phase variations (windowed differences under the histogram show background phase variation and edge effects)
**FIGURE S2** Determination of the order in which voxels are unwrapped, illustrated for a 4 × 4 image. Unwrapping proceeds from the gray seed voxel in the order indicated by the blue number on each arrow, following the order of the quality values (in black) of the edges of the voxels that have already been unwrapped
**FIGURE S3** The effect of unwrapping errors on field maps and the distortion correction of EPI. Errors unwrapping EPI phase data yield erroneous field map values (at yellow arrows) and lead to corruption of the magnitude (red arrows)
**TABLE S1** Percentage of erroneous voxels for BEST PATH and ROMEO unwrapping results for the complex topography with no noise and 10% noise at three TEs (PRELUDE did not complete). Note: Voxels with 2πn phase differences from the ground‐truth phase (where n is an integer) were counted as erroneous.

## Data Availability

The code that supports the findings of this study is openly available in GitHub at https://github.com/korbinian90/ROMEO.jl/tree/publication. We plan continuous support and development of the software with the newest version available in https://github.com/korbinian90/ROMEO. The MRI data are available in Harvard Dataverse at https://dataverse.harvard.edu/dataverse/ROMEO.
