# Supplementary material for: Phase unwrapping with a rapid opensource minimum spanning tree algorithm (ROMEO)
Source: Magn Reson Med. 2020 Oct 26;85(4):2294–308. doi: 10.1002/mrm.28563 (PMC7821134; doi:10.1002/mrm.28563)
Supplement: Supplementary file 1 — FIGURE S1 A comparison of the path‐based method (ROMEO [rapid opensource minimum spanning tree algorithm]) and Laplacian unwrapping. The path‐based method restores the simulated ground‐truth phase from the wrapped phase, yielding exactly the true phase value in every voxel. The Laplacian method removes wraps but introduces phase offsets and background phase variations (windowed differences under the histogram show background phase variation and edge effects) FIGURE S2 Determination of the order in which voxels are unwrapped, illustrated for a 4 × 4 image. Unwrapping proceeds from the gray seed voxel in the order indicated by the blue number on each arrow, following the order of the quality values (in black) of the edges of the voxels that have already been unwrapped FIGURE S3 The effect of unwrapping errors on field maps and the distortion correction of EPI. Errors unwrapping EPI phase data yield erroneous field map values (at yellow arrows) and lead to corruption of the magnitude (red arrows) TABLE S1 Percentage of erroneous voxels for BEST PATH and ROMEO unwrapping results for the complex topography with no noise and 10% noise at three TEs (PRELUDE did not complete). Note: Voxels with 2πn phase differences from the ground‐truth phase (where n is an integer) were counted as erroneous. [file MRM-85-2294-s001.docx]

***Supporting Information Figure S1: Loss of quantitative phase information with Laplacian unwrapping***

A sphere with 10% noise (expressed as a percentage of the phase at each echo time) was simulated, as in Ref (1), with the addition of a square structure with abrupt edges (see Figure S1, ‘True Phase’), and wraps were introduced. This image was unwrapped using ROMEO (as an example of a path-following method) and the Laplacian method (2). ROMEO restored the ground truth values exactly (see difference image and histogram). The Laplacian method removed wraps but introduced different offsets in each part of the image (peaks in the histogram of the difference between the ground truth phase and the Laplacian), and background phase variations in each part of the image, apparent in the breadth of each peak. Any simple relationship between the Laplacian-unwrapped and true phase values is lost, rendering the unwrapped phase useless for most quantitative application (e.g. B_0_ mapping, measuring flow velocity, temperature).

| ***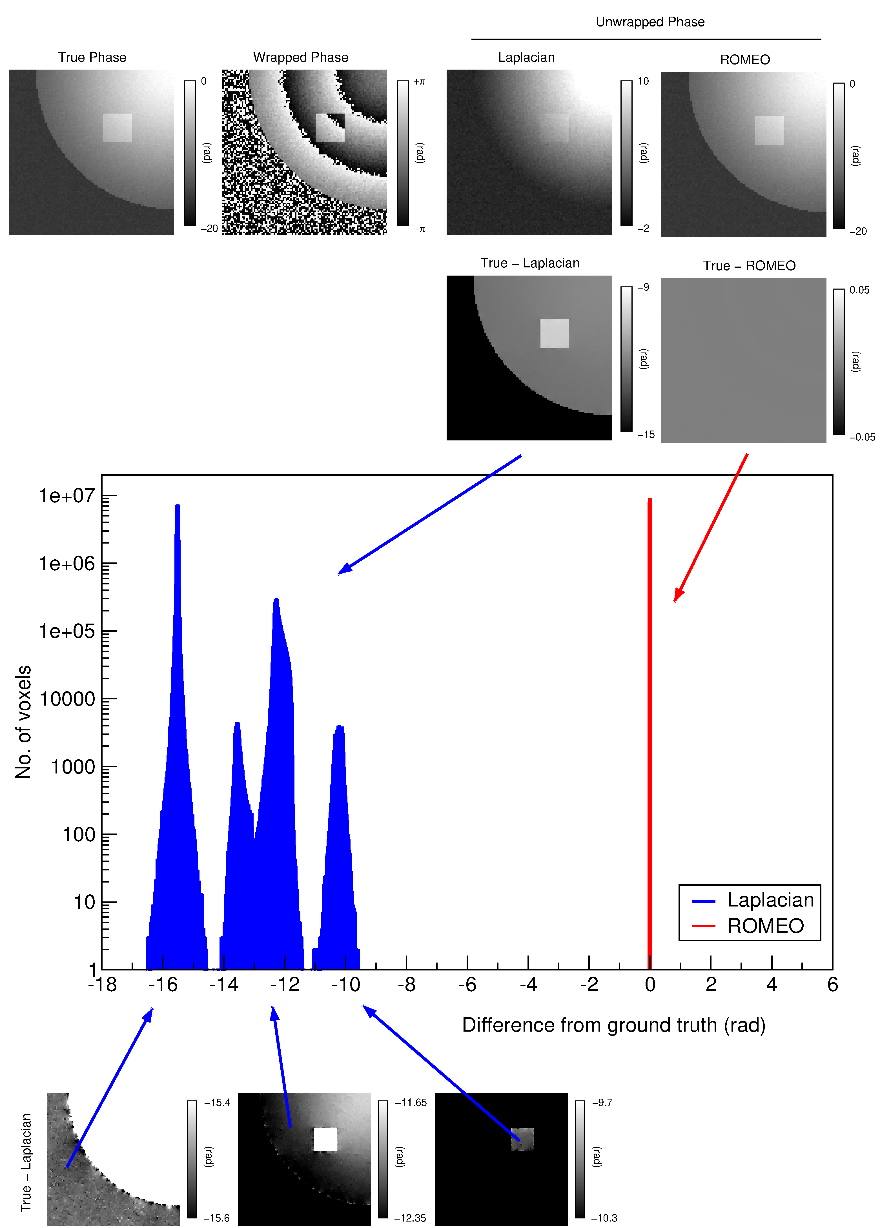*** |
| --- |
| ***Figure S1****: A comparison of path-based method (ROMEO) and Laplacian unwrapping. The path-based method restores the simulated ground truth phase from the wrapped phase, yielding exactly the True Phase value in every voxel. The Laplacian method removes wraps but introduces phase offsets and background phase variations (windowed differences under the histogram show background phase variation and edge effects).* |

***Supporting Information Figure S2: Explanation of the minimum spanning tree, or order of voxel unwrapping***

The order in which voxels are unwrapped is illustrated for a notional image of 4x4 voxels in Figure S2. Unwrapping begins at the seed voxel shaded grey, which is one of a two voxels having the edge with the highest quality value (0.98). In ROMEO indexing from left to right and top to bottom is chosen, therefore voxel to the left of the edge with value 0.98 is the seed voxel. The edge values of that voxel are entered into the queue: (0.28,0.98,0.96,0.32). The first voxel to be processed is that connected to the seed voxel via the edge with the highest quality value, 0.98 (arrow 1). If the phase difference between these two voxels is > π, this voxel is unwrapped by adding or subtracting 2π (as explained in the Methods) and is marked as ‘visited’, the value 0.98 is removed from the queue, and the values of the edges of the newly-connected voxel (0.65,0.68,0.72) are added, such that the queue now contains the values (0.28,0.96,0.32,0.65,0.68,0.72). The next highest value in the queue is 0.96, defining the next voxel to be unwrapped (arrow 2). That voxel is unwrapped and marked as visited, the value 0.96 is removed from the queue and edges of the newly unwrapped voxel (0.61,0.84,0.65) added to it. In case next highest quality value in the queue connects two visited voxels it is removed from the queue. The algorithm proceeds in this way until all voxels have been considered. Note that this explanation is based on quality values described in the text, but (for consistency with prior phase unwrapping literature and graph theory), the algorithm actually uses cost values, which are 1-quality values (so low cost values represent reliable connections), and selects the lowest cost value in the queue. The same order results. In the data presented in this manuscript the algorithm is performed in 3D by considering 6 neighbours per voxel instead of 4.

| ***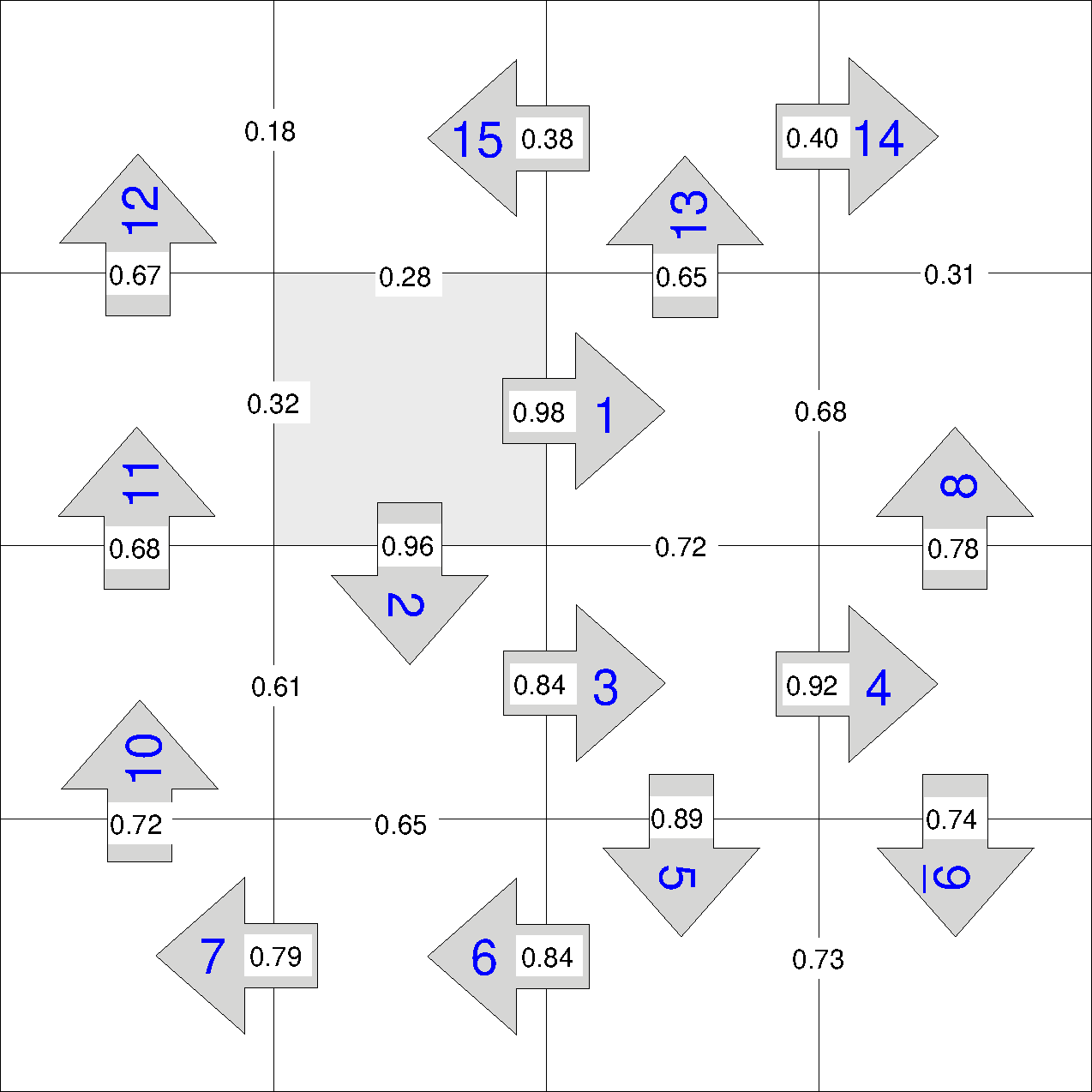*** |
| --- |
| ***Figure S2:*** *Determination of the order in which voxels are unwrapped, illustrated for a 4x4 image. Unwrapping proceeds from the grey seed voxel in the order indicated by the blue number on each arrow, following the order of the quality values (in black) of the edges of the voxels which have already been unwrapped.* |

***Supporting Information Figure S3: The effect of unwrapping errors on the correction of distortions in EPI.***

EPI volumes (see Table 1) unwrapped with PRELUDE, BEST PATH and ROMEO were converted to B_0_ field maps by division by the echo time. These were applied to the corresponding magnitude EPI images using FSL’s fugue (3) (with the “-w” option to apply a forward warp, as the field maps were themselves distorted), dynamically correcting the distortions in the time series (4). Two selected slices are illustrated for the first time point for Patient 5 (the same data illustrated in Figure 5 in the main manuscript). Distortion in magnitude images (Figure S3, left column) is well corrected (right 3 columns), other than at the locations indicated by red arrows, where unwrapping errors in PRELUDE and BEST PATH led to gross errors in field estimation (yellow arrows) and unphysical voxel shifts.

| ***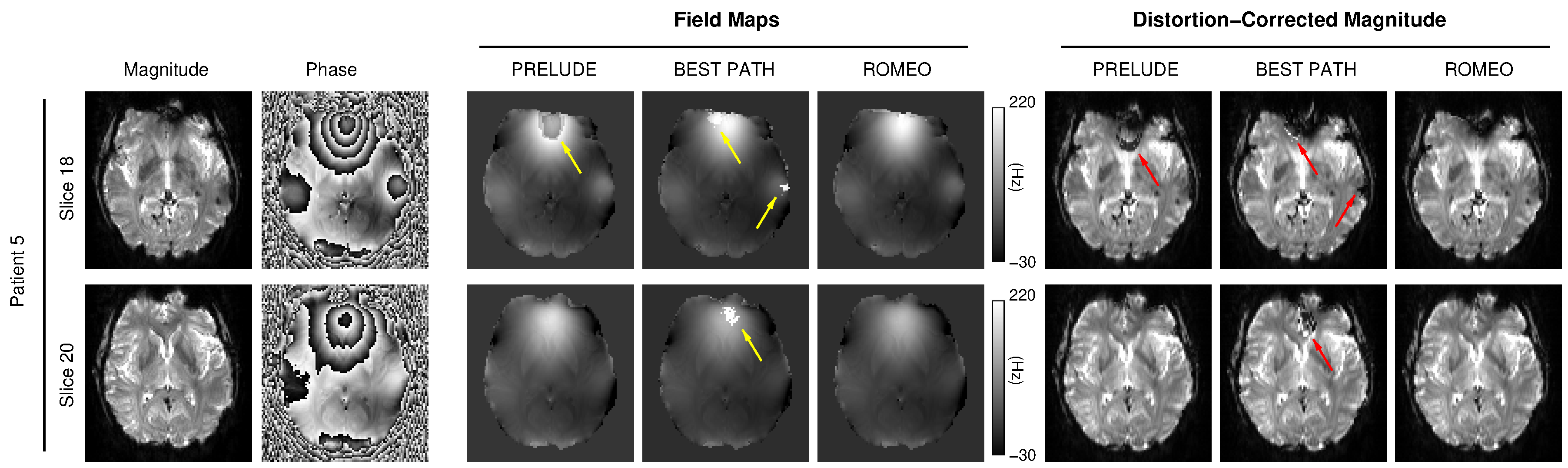*** |
| --- |
| ***Figure S3:*** *The effect of unwrapping errors on field maps and the distortion correction of EPI. Errors unwrapping EPI phase data yield erroneous field map values (at yellow arrows) and lead to corruption of the magnitude (red arrows).* |

|  |  | **No. of erroneous voxels [%]** | |
| --- | --- | --- | --- |
| Noise Percentage | TE [ms] | BEST PATH | ROMEO |
| 0 | 4.0 | 0.00 | 0.00 |
|  | 8.0 | 1.99 | 0.00 |
|  | 10.0 | 7.52 | 0.00 |
| 10 | 4.0 | 0.00 | 0.00 |
|  | 8.0 | 2.97 | 0.00 |
|  | 10.0 | 8.91 | 0.10 |

**Table S1**. The percentage of erroneous voxels for BEST PATH and ROMEO unwrapping results for the complex topography with no noise and 10% noise at 3 echo times (PRELUDE did not complete). Voxels with 2πn phase differences from the ground truth phase (where n is an integer) were counted as erroneous.

**References**

1. Robinson S, Schödl H, Trattnig S. A method for unwrapping highly wrapped multi-echo phase images at very high field: UMPIRE. Magn. Reson. Med. 2014;72:80–92 doi: 10.1002/mrm.24897.

2. Volkov VV, Zhu Y. Deterministic phase unwrapping in the presence of noise. Opt Lett 2003;28:2156–8.

3. Jenkinson M. Improving the registration of B0-disorted EPI images using calculated cost function weights. Tenth Int. Conf. Funct. Mapp. Hum. Brain 2004.

4. Dymerska B, Poser BA, Barth M, Trattnig S, Robinson SD. A method for the dynamic correction of B0-related distortions in single-echo EPI at 7T. NeuroImage 2018;168:321–331 doi: 10.1016/j.neuroimage.2016.07.009.
